# Physical workload and cardiopulmonary parameters in relation to individual capacity of bulk waste workers – a cross-sectional field-study

**DOI:** 10.1186/s12995-023-00389-z

**Published:** 2023-12-15

**Authors:** Alexander Michael Kraft, Marcial Velasco Garrido, Robert Herold, Volker Harth, Alexandra Marita Preisser

**Affiliations:** Institute for Occupational and Maritime Medicine, University Medical Center Hamburg-Eppendorf, Seewartenstrasse 10, 20459 Hamburg, Germany

**Keywords:** Heavy physical work, Manual work, Relative aerobic strain, Heart rate reserve, VT1, Anaerobic threshold, Physical capacity, Bulk waste collection, Waste management

## Abstract

**Purpose:**

Waste collection is considered particularly heavy work, although no previous study has yet investigated the strain of bulk waste collection. The aim of this study is to determine the workload of bulk waste workers in practice.

**Method:**

We conducted a cross-sectional field-study. Fourteen male volunteers from the bulk waste collection of the municipal sanitation department in Hamburg, Germany, were included. Performance was determined by cardiopulmonary exercise testing under laboratory conditions. During the shift, each worker was accompanied by a researcher, and heart rate (HR) was recorded under field conditions using an HR watch with a belt system. We examined mean HR, relative heart rate (RHR), relative aerobic strain (RAS), calculated oxygen uptake ($\mathrm{\overset{.}{V}O}_2$)  and individual ventilatory threshold 1 (VT1) as parameters of workload during their daily work.

**Results:**

During the shift, HR was scaled: 102 bpm (SD 10.2), RHR: 36.9%, $\mathrm{\overset{.}{V}O}_2$: 1267 ml/min (SD 161), RAS: 49.4% (SD 9.3), and $\mathrm{\overset{.}{V}O}_2$ in relation to VT1: 75% (SD 18.5). There was no significant difference between oxygen consumption during the main task of lifting and carrying bulky waste and the individual $\mathrm{\overset{.}{V}O}_2$ at VT1.

**Conclusion:**

Although the burden of the main task of lifting and carrying bulky waste is very high (at VT1 for more than 3 h), interruptions from other tasks or formal breaks spread the burden over the entire shift. The total workload exceeded most recommendations in the literature across the different work periods. However, the total burden remains below VT1, the only parameter that takes individual endurance performance into account. We recommend again VT1 as an individual upper limit for prolonged occupational work.

**Supplementary Information:**

The online version contains supplementary material available at 10.1186/s12995-023-00389-z.

## Introduction

Chronic overload in professions with long-term exposure to heavy work has been shown to be associated with cardiovascular disease and all-cause mortality [1]. In the last decade, little scientific attention has been paid to cardiovascular strain in waste management and collection work, despite evidence that this activity is generally physically demanding [2–6]. To our knowledge, no previous study has specifically examined the work of bulk waste collection, which is completely different from conventional household garbage collection. In Hamburg, bulky waste collectors drive from customer to customer to pick up bulky waste (mainly old mattresses, furniture, or large electrical appliances, such as washing machines, refrigerators, etc.) from private households and company premises. The work involves lifting and carrying heavy and difficult-to-handle objects. Thus, high levels of strain and health consequences are also to be expected for bulk waste workers. So far, there is little scientific evidence for this profession to develop targeted preventive interventions.

Maintaining employment in physically demanding professions is largely determined by the balance between individual physical fitness and occupational physical demands [7]. To estimate a person’s cardiovascular workload and physical demands during a workday, there are two commonly used parameters: relative heart rate (RHR), measured as percentage of the heart rate reserve (HRR and %HRR, resp.), and relative aerobic strain (RAS), defined as oxygen consumption ($\mathrm{\overset{.}{V}O}_2$) during work relative to the individual’s maximum $\mathrm{\overset{.}{V}O}_2$, $(\%{\overset.{\mathrm V}\mathrm O}_{2,\max})$. In general, higher values of %HRR and $\%{\overset.{\mathrm V}\mathrm O}_{2,\max}$ indicate higher physical demands during an activity. There have been various recommendations for acceptable workload limits based on these parameters. For example, it has been recommended that the maximum allowable heart rate during an 8-h workday for general manual work should be 33% of HRR [8]. Regarding oxygen consumption, a maximum RAS between 30 and 35% has been proposed for manual labour [9–13]. It is controversial whether a higher RAS (e.g. 40%-50% ${\overset.{\mathrm V}\mathrm O}_{2,\max}$) might be acceptable if sufficient rest periods are granted or the duration of the task is limited [14–17]. According to more recent publications, a maximum acceptable RAS of 33–40% ${\overset.{\mathrm V}\mathrm O}_{2,\max}$ seems too low, since this value does not represent a physiologically reasonable limit in relation to thresholds such as the ventilatory threshold (VT1) or the lactate turning point 1 (LTP1) [3, 18–20]. The VT1 and LTP1 allow us to identify the individual turning point from aerobic to anaerobic metabolism and have therefore been proposed as indicators of heavy workload [20–26]*.*

The aim of our study was to determine the degree of physical workload and cardiovascular strain of bulk waste collectors using RHR, RAS and $\mathrm{\overset{.}{V}O}_2$ at VT1 $({\overset.{\mathrm V}\mathrm O}_{2,{\mathrm{VT}1}})$ and to compare it to the thresholds proposed in the literature.

## Methods

### Study design

We conducted a cross-sectional field-study. Participants underwent a preliminary medical examination (PME) at our clinic and were observed on site throughout the collection tour for one working day. The study was part of a broader occupational and ergonomic field-study that assessed the working conditions and strain of bulk waste collectors with qualitative interviews, real-life observation, and objective strain measurements [27].

### Selection of study population

Participants were recruited among workers of the bulk waste collection department (*n* = 105, all male) of the municipal waste collection services in the City of Hamburg, Germany. The collectors and drivers participated voluntarily in the respected study. Fourteen tours were selected by the company's tour planners and the safety engineer. The sampling method was predefined by the ergonomic study with the objective of representing a variety of collection tours. Participants had the option to drop out at any time. Participation was rewarded with a day off.

To be invited to the PME, participants had to be ≥ 18 years old and provide written consent for cardiopulmonary exercise testing (CPX). In order to participate in the field-study, an interpretable and non-pathological baseline CPX had to be obtained at our clinic. Participants using HR-modifying drugs were excluded from the field-study.

### Preliminary medical examination

The purpose of the PME was to identify pre-existing medical conditions or medications that would affect HR and thus complicate the interpretation of the field measurement and to provide baseline data for the extrapolation of $\mathrm{\overset{.}{V}O}_2$ from HR measured in the field.

The PME was performed between February and June 2018 at our clinic and consisted of a medical history, clinical examination, 12-lead resting-ECG, and CPX. Additionally, spirometry and body plethysmography (MasterScreen™Body by JAEGER™/CareFusion Germany 234 GmbH) were performed. Forced expiratory capacity in one second (FEV1), Tiffeneau-Index ((FEV1/FVC) × 100%), and airway resistance (sRt) were measured to assess the potential presence of obstructive pulmonary disease. Forced vital capacity (FVC) and total lung capacity (TLC) were also recorded to identify any signs of a restrictive lung disease. The results were interpreted according to current guidelines and reference values [28–30].

CPX was performed according to current recommendations by Meyer et al. (2018) [31] using a cycle ergometer (Vyntus CPX device from Vyaire, Höchberg). The device was volume- and gas-calibrated before each CPX. The CPX protocol starts with a rest phase of 2 min, followed by a reference phase of 2 min with unloaded pedalling, then pedalling with continuously increasing load (the test phase), and a subsequent recovery phase of 3 to 5 min. The maximum physical load should be reached after 10 ± 2 min (T_test phase_). The load increase was previously determined considering the age, body weight, and training status of each individual and varied between 15, 20, or 25 W/min. CPX was terminated according to the criteria proposed by Meyer et al. [31] or when the subject could no longer maintain speed. Oxygen uptake $(\mathrm{\overset{.}{V}O}_2)$, carbon dioxide production $({\overset.{\mathrm V}\mathrm{CO}}_2)$, and heart rate (HR) ECG were continuously recorded. As a criterion for reaching maximal oxygen uptake $({\overset.{\mathrm V}\mathrm O}_{2,\max})$, we chose a limit of > 1.15 of the maximal respiratory exchange rate (RER) and a flattening of the $\mathrm{\overset{.}{V}O}_2$ curve. VT1 was determined by the V-slope method as the first disproportionate increase in ${\overset.{\mathrm V}\mathrm{CO}}_2$ relative to $\mathrm{\overset{.}{V}O}_2$ [24]. The endurance capacity was defined with ${\mathrm{\overset{.}{V}O}}_{2,\mathrm{VT}1}{\%{\overset.{\mathrm V}\mathrm O}}_{2,\max,\mathrm{pred}}$ according to Kroidl et al. (2014) (< 40% pathological, 40–49% untrained, 50–59% normal, 60–80% athletic) [32].

### Field observations

Each individual worker was accompanied by a researcher throughout the collection tour. The duration of the tasks as well as the conditions of collection were documented using a standardised data collection sheet. The amount of compacted waste was documented to compare with historical data from this department’s collection tours from 2005 to 2018.

### Assessment of physical demand

During the bulk waste collection tour, the participants' heart rates were recorded in 5-s intervals using an HR watch with a belt system (Polar WearLink® W.I.N.D. transmitter, Polar RS°800™ and Polar RS 800cx™, Polar Electro Oy, Kempele, Finland). To reduce the influence of possible artefacts, 1-min averages were calculated (HR_field_). If HR recording was interrupted, the data gaps were not included in the analysis; all parameters refer to the actual recording time.

The resting HR of the participants (HR_rest_) was defined as the lowest HR recorded in the resting ECG, CPX, or HR_field_ values. The maximum heart rate of the participant (HR_max_) was defined as the highest HR measured in the CPX or HR_field_. Individual heart rate reserve (HRR) was calculated as the difference between individual HR_max_ and HR_rest_. The recording began just before the workers left the depot and ended when they returned to the depot after the collection tour, indicating the duration of the shift (T_shift_). T_shift_ was further differentiated into summed phases of driving (T_driving_), the main task of bulky waste pickup from the customer (T_C_), and a lunch break (T_break_). For each minute during T_shift_, the RHR (RHR_field_) was calculated individually using the formula: RHR_field_ = (HR_field_ – HR_rest_)/HRR * 100%. We calculated the mean HR for the durations of T_shift_ (HR_shift_) and T_C_ (HR_C_) and the mean RHR for shift (RHR_shift_) and collection (RHR_C_)) for each subject. We also calculated the individual recommended maximal RHR for the whole shift (RHR_shift,rec_) and for the collection time (RHR_C,rec_) according to the formula proposed by Wu & Wang (2002) T [hours] = 26.12e^-4,81*(RHR) [%] [17] with T_shift_ orT_C_ respectively.

The CPX performed in PME was used to describe the linear relationship between HR and $\mathrm{\overset{.}{V}O}_2$ for each individual. We calculated correlation coefficients (r) and linear regression equations between $\mathrm{\overset{.}{V}O}_2$ and HR to estimate field oxygen uptake $({\overset.{\mathrm V}\mathrm O}_{2,\mathrm{field}})$ from HR_field_ [3, 16, 33, 34]. For each minute during T_shift_, the RAS (RAS_field_) was calculated individually using the formula:  ${\mathrm{RAS}}_{\mathrm{field}}\;={\overset.{\mathrm V}\mathrm O}_{2,\mathrm{field}}/\;{\overset.{\mathrm V}\mathrm O}_{2,\max\;}\ast\;100\%$. Using the values of the ${\overset.{\mathrm V}\mathrm O}_{2,\mathrm{field}}$ and the RAS_field_, we calculated the mean values for $\mathrm{\overset{.}{V}O}_2$ $({\overset.{\mathrm V}\mathrm O}_{2,\mathrm{shift}},{\overset.{\mathrm V}\mathrm O}_{2,\mathrm C})$ and the RAS (RAS_shift_ and RAS_C_) for the durations of T_shift_ and T_C_ for each subject. We also calculated the individual recommended RAS (RAS_shift,rec_, RAS_C,rec_) according to Wu & Wang (2002) with the recorded times of T (T_shift_ and T_C_) according to the following equation: T [hours] = 95.33e^-7.28*(RAS) [%] [17].

### Statistical analysis

Statistical analyses were performed using IBM® SPSS® Statistics (IBM Corp., released 2015. IBM SPSS Statistics for Windows, Version 23.0. Armonk, NY, USA) and R: A Language and Environment for Statistical Computing, 2021 [35].

We calculated the individual differences between the workload parameters measured in the field and their recommended values, (RHR_shift_ – RHR_shift,rec_; RAS_shift_ – RAS_shift,rec_; RHR_C_ – RHR_C,rec_). We also calculated the difference between oxygen consumption extrapolated from field measurements and oxygen consumption at the ventilatory threshold measured in CPX $\left(\mathrm{\overset{.}{V}O}_{2,\text{shift}}-\mathrm{\overset{.}{V}O}_{2,\text{VT1}},\,\mathrm{\overset{.}{V}O}_{2,\text{C}}-\mathrm{\overset{.}{V}O}_{2,\text{VT1}}\right)$. The normality of the distributions' differences was examined using the Shapiro–Wilk-Test [36]. We performed paired t-tests to determine whether field measurements and recommended values differed. The aim is to determine whether workloads were above or below recommended values. To account for multiple comparisons, we used the Bonferroni-Holm method [37].

In order to determine whether our results differ from similar studies [3–6, 13, 20], we additionally calculated the difference between the means of our study and the means reported in the mentioned publications with their corresponding 95% confidence intervals (Additional file 3: Other studies vs. our results). The significance level was set at *p* = 0.05.

## Results

**Fig. 1:**
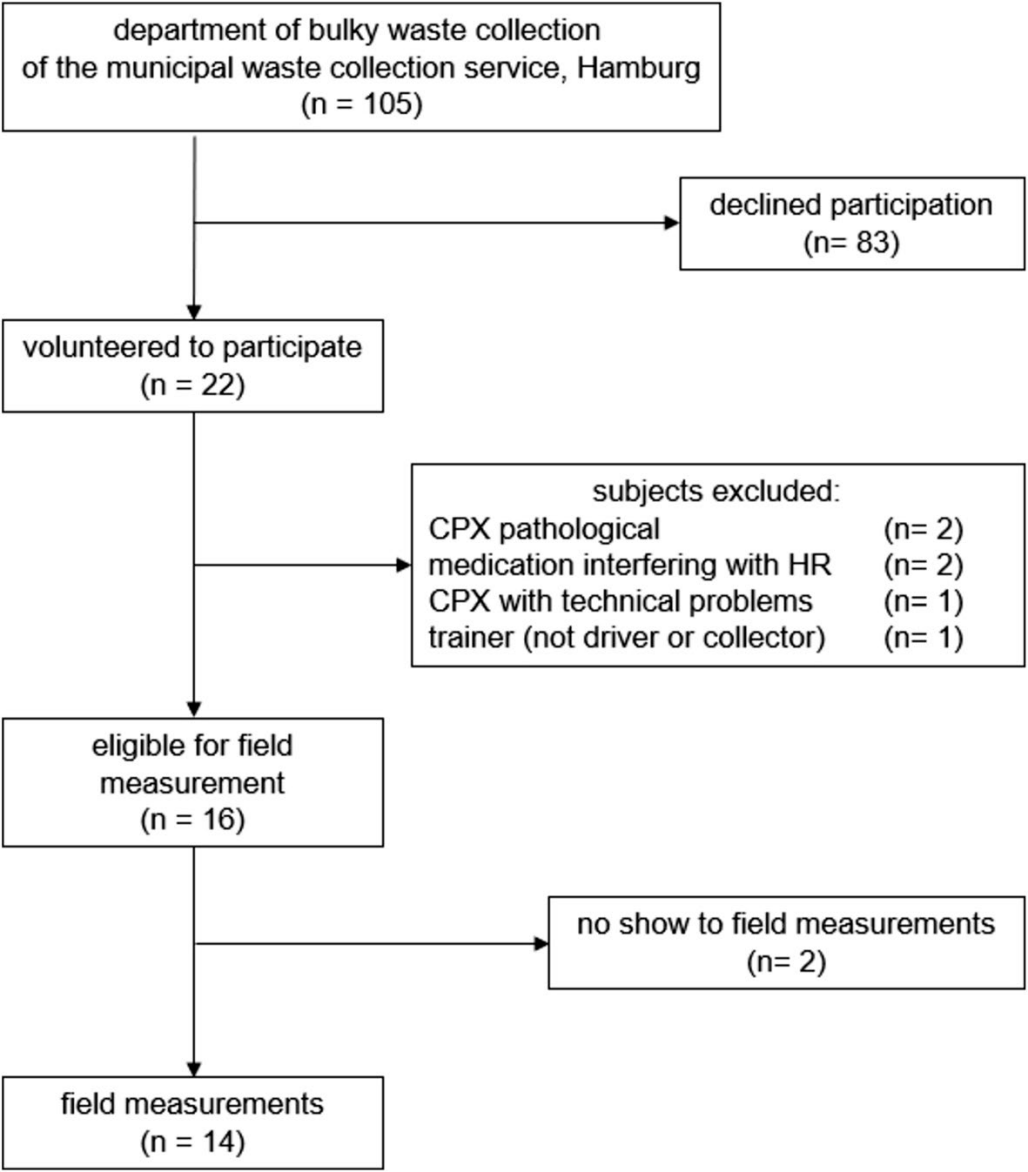
Study population *CPX* Cardiopulmonary exercise testing

Twenty-two collectors volunteered to participate in the PME. Six were excluded from the field measurements for different reasons, and two dropped out of the study (Fig. 1). Field measurements were carried out on 14 volunteers, whose data is included in the analysis.

### Preliminary medical examination

The characteristics of the study participants are shown in Table 1. The participants were, on average, 42.6 years old (SD 10.6). According to the WHO definition, 21.4% of the subjects had a normal body weight, 35.7% were overweight (BMI ≥ 25) and 42.9% were obese (BMI ≥ 30)*.* Of the 14 subjects included, 35.7% were active smokers, 28.6% were former smokers, and 35.7% had never smoked. Three subjects (21.4%) showed airway obstruction with a Tiffeneau-Index below the lower limit of normal; two of them were active smokers and the other had never smoked. However, airway resistance (sRt) was normal (< 1.18 kPa*s) in all subjects (Table 2). In spirometry, none of the subjects showed a restrictive pulmonary disease. In CPX, a mean maximum respiratory exchange rate (RER_max,mean_) of 1.5 and a mean ${\overset.{\mathrm V}\mathrm O}_{2,\mathrm{VT}1}/{\overset.{\mathrm V}\mathrm O}_{2,\max,\mathrm{pred}}$ of 61.2% (SD 14.1) were achieved, indicating athletic endurance levels with below average ${\overset.{\mathrm V}\mathrm O}_{2,\max}\%{\overset.{\mathrm V}\mathrm O}_{2,\max,\mathrm{pred}}$ of 90.5% (SD 13) [32]. Regarding maximum power output (P_max_/P_max,pred_: 104% SD 14.4), the subjects performed above the predicted values, see Table 2. We were able to demonstrate a linear correlation between HR and $\mathrm{\overset{.}{V}O}_2$ with a mean r of 0.95 (SD 0.04) (Table 2); this made it possible to calculate $\mathrm{\overset{.}{V}O}_2$ from the recorded HR data during the field measurements [3, 17, 33, 34].

**Table 1:** Characteristics of the study population

*n*=*14*		**mean**	**SD**	**median**	**min**	**max**
**Age**	[years]	42.6	10.6	44.6	25.6	57.1
**Height**	[cm]	178	5.5	178	167	185
**Weight**	[kg]	94	16.1	90	76	123
**BMI**	[kg/m^2^]	29.7	5.4	28.9	23.9	42
**Years working in bulky waste disposal**	[years]	14.9	11.6	14	3	38

*PME* Preliminary medical examination, *CPX* Cardiopulmonary exercise test, *BMI* Body mass index, *SD* Standard deviation

**Table 2:** Results of lung function and cardiopulmonary exercise testing (CPX)

***n* = 14**		**mean**	**SD**	**median**	**min**	**max**
**FVC**	[L]	5.23	0.81	5.04	4.02	6.71
**FVC**_**%pred**_	[%]	101.5	11.05	103	79	115
**FEV1**	[L]	4.05	0.63	4.06	3.03	5.55
**FEV1**_**%pred**_	[%]	97.2	10.7	94.6	83.0	115.0
**Tiffeneau-Index**	[%]	78.1	5.12	77.4	70.4	85.0
**TLC**	[L]	7.11	0.80	7.07	5.71	8.94
**TLC**_**%pred**_	[%]	99.5	10.33	100.0	81.8	116.1
**sR**_**tot**_	[kPa*s]	0.67	0.26	0.62	0.36	1.08
**T**_**test phase**_	[min:sec]	09:46	01:45	09:37	06:09	12:34
**P**_**max**_	[W]	219	43.6	217	138	280
**P**_**max**_**%P**_**max,pred**_	[%]	104	14.4	101	74	127
**P**_**max**_**/BW**	[W/kg]	2.36	0.47	2.41	1.18	3.18
${\overset{\boldsymbol.}{\mathbf V}\mathbf O}_{\mathbf2\boldsymbol,\mathbf m\mathbf a\mathbf x}$	[ml/min]	2606	463	2570	1818	3260
${\overset{\boldsymbol.}{\mathbf V}\mathbf O}_{\mathbf2\boldsymbol,\mathbf m\mathbf a\mathbf x}\boldsymbol\%{\overset{\boldsymbol.}{\mathbf V}\mathbf O}_{\mathbf2\boldsymbol,\mathbf m\mathbf a\mathbf x\boldsymbol,\mathbf p\mathbf r\mathbf e\mathbf d}$	[%]	90.5	13	91.3	57.2	109
${\overset{\boldsymbol.}{\mathbf V}\mathbf O}_{\mathbf2\boldsymbol,\mathbf m\mathbf a\mathbf x}\boldsymbol/\mathbf{BW}$	[ml/kg]	28	4.6	27	20.8	36.3
**RER**_**max**_		1.5	0.16	1.51	1.19	1.82
**P**_**VT1**_	[W]	136	39.3	123	82	210
**P**_**VT1**_**%P**_**max**_	[%]	61.7	10.5	60.3	43.2	81.5
${\overset{\boldsymbol.}{\mathbf V}\mathbf O}_{\mathbf2\boldsymbol,\mathbf V\mathbf T\mathbf1}$	[ml/min]	1758	409	1680	991	2517
${\overset{\boldsymbol.}{\mathbf V}\mathbf O}_{\mathbf2\boldsymbol,\mathbf V\mathbf T\mathbf1}\boldsymbol\%{\overset{\boldsymbol.}{\mathbf V}\mathbf O}_{\mathbf2\boldsymbol,\mathbf m\mathbf a\mathbf x\boldsymbol,\mathbf p\mathbf r\mathbf e\mathbf d}$	[%]	61.2	14.1	60.7	41.0	88.7
**HR**_**rest**_	[bpm]	65.9	10.1	67	52	82
**HR**_**max**_	[bpm]	161	13,1	162	138	182
**HR**_**max**_**%HR**_**max,pred**_	[%]	89.8	5.92	89	80	99
**HR**_**VT1**_	[bpm]	120	9.31	119	106	139
**HR**_**VT1**_**%HR**_**max**_	[%]	74.34	7.07	73.8	60.9	89.3
**r**		0.95	0.04	0.95	0.83	0.99

*FVC* Forced vital capacity, *FVC%pred* % of the predicted FVC, *FEV1* Forced expiratory capacity in one second*, FEV1*_*%pred*_ % of the predicted FEV1, *Tiffeneau-Index* (FEV1/FVC) × 100%, *TLC* Total lung capacity, *TLC*_*%pred*_ % of the predicted TLC, *sR*_*tot*_ Total specific airway Resistance*, T*_*test phase*_ Duration of pedaling with increasing load during CPX,* P* Power output, *P*_max_ Maximum power output, *P*_max_%*P*_max,pred_ P_max_ relative to predicted values of maximum power output (P_max,pred_), *P*_*max*_*/BW *P_max_ relative to the body weight (BW), ${\overset.VO}_{2,max}$ Maximum oxygen consumption, ${\overset{\cdot}{V}O}_{\textit 2,\textit{max}}\%{\overset{\cdot}VO}_{\textit 2,\textit{max,pred}}\;{\overset.{\text V}\mathrm O}_{2,\max}$  relative to predicted values of maximum oxygen consumption $({\overset.{\mathrm V}\mathrm O}_{2,\max,\mathrm{pred}})$, ${\overset.VO}_{2,max}/BW$ ${\overset.VO}_{2,max}$ relative to the BW, *RER*_*max*_ Maximum respiratory exchange rate, *P*_VT1_ Power output at the ventilatory threshold 1 (VT1), *P*_VT1_%*P*_max _P_VT1_ relative to *P*_max,_ ${\overset.VO}_{2,VT1}$ Oxygen consumption at VT1 ${\overset{\cdot}{\textit V}\textit{O}}_{\textit 2,\textit V\textit T\textit 1}\%{\overset{\cdot}{\textit V}\textit O}_{\textit 2,\textit{max,pred}}\;{\overset.{\text V}\text O}_{2,\text{VT}1}$  relative to ${\overset.{\mathrm V}\mathrm O}_{2,\max,\mathrm{pred}}$ as a marker for endurance capacity according to [32], *HR*_*rest*_ resting heart rate, *HR*_*max*_ Maximum heart rate, *HR*_*max*_*%HR*_*max,pred*_ HR_max_ relative to predicted values of maximum heart rate (HR_max,pred_), *HR*_*VT1*_ Heart rate at VT1, *HR*_*VT1*_*%HR*_*max*_ HR_VT1_ relative to HR_max_, *SD* Standard deviation

### Field observations

Field observations were made during 14 collection tours. Each subject was accompanied by a researcher. The tours began with an average one-hour drive to the first place of work (60.79 min SD 13.17). The collection tours consisted of 7 to 17 different stops in single-family homes, basements, flat buildings with or without lifts, or industrial premises. HR measurements were accidentally interrupted once by subjects 4 and 11 for 36 and 7 min, respectively (Additional file 4: Heartrate measurements in the field: subject 4 (03:50 – 04:26 h) and 11 (05:54 – 06:01 h)). The total quantity of compressed bulky waste collected during the field observations ranged from 2.43 to 5.31 metric tons (t), with an average of 3.9 t collected by five workers per shift. The driving intervals (T_int,driving_) lasted on average 14.8 min (SD 4.1), and the intervals of handling bulky waste at each stop (T_int,C_) lasted on average 16.4 min (SD 4.0). Approximately halfway through the shift, a 30-min lunch break was provided. See also the figures in Additional file 4: Heartrate measurements in the field showing HR_field_ over the duration of T_shift_ for each individual.

**Fig. 2:**
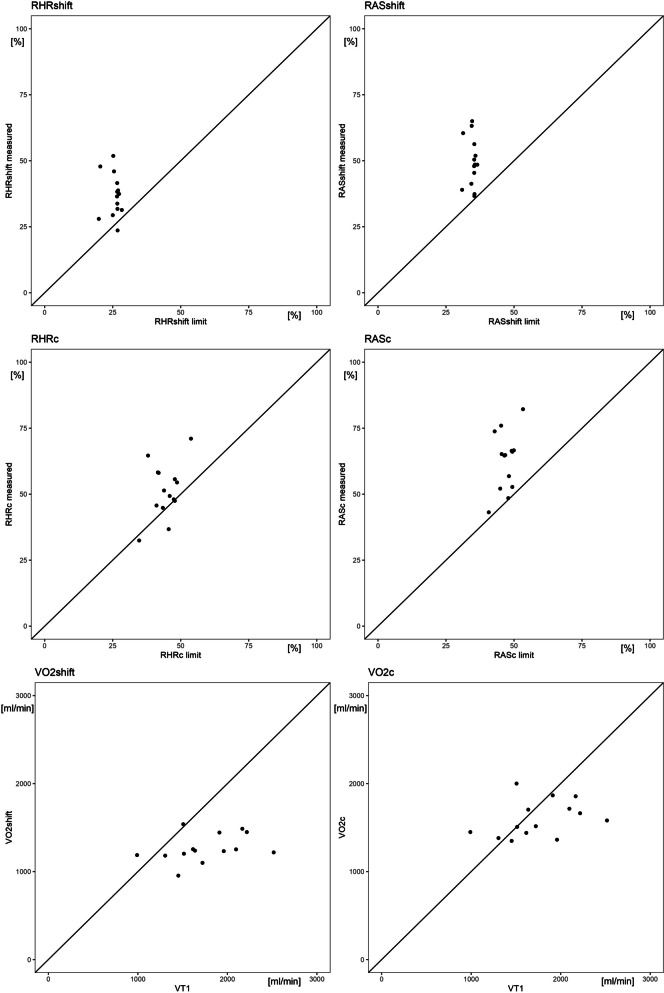
The individual measurements of ${\mathrm{RHR}}_{\mathrm{shift}}\;\mathrm{and}\;{\mathrm{RHR}}_{\mathrm C},\;{\mathrm{RAS}}_{\mathrm{shift}},\;{\mathrm{RAS}}_{\mathrm C},\;{\overset.{\mathrm V}\mathrm O}_{2,\mathrm{shift}}{\overset.{\mathrm V}\mathrm O}_{2,\mathrm C}$ in relation to the recommended limits for prolonged occupational work according to [17] and ${\dot{\mathrm{V}}\mathrm{O}}_{2,VT1}$ ${RHR}_{shift}$ Individual mean relative heart rate (RHR) during T_shift_, *RHR*_*shift,rec*_ The calculated maximum recommended RHR for T_shift_ [17], *RHR*_*C*_ Individual RHR during T_C_, *RHR*_*C,rec*_ The calculated maximum recommended RHR for T_C_ [17], *RAS*_*shift*_ Individual mean relative aerobic strain (RAS) during T_shift_, *RAS*_*shift,rec*_ The calculated maximum recommended RAS for T_shift_ [17], *RAS*_*C*_ Individual RAS during T_C_, *RAS*_*C,rec*_ The calculated maximum recommended RAS for T_C_ [17], ${\overset.VO}_{2,shift}$ Individual mean oxygen consumption ($\mathrm{\overset{.}{V}O}_2$) during the individual shift, ${\overset.VO}_{2,VT1}$ Individual oxygen consumption at ventilatory threshold (VT1), ${\overset.VO}_{2,C}$ Individual mean $\mathrm{\overset{.}{V}O}_2$ during the individual total time collecting manually bulky waste from the premises of the customer summed over the duration of T_shift_ (T_C_)

We observed an average HR_shift_ of 102 bpm (SD 10.2), equivalent to 85.7% (SD 10.6) of HR at VT1 and an average RHR_shift_ of 36.9% (SD 8.0). While collecting waste, the subjects showed a mean HR_C_ of 116 (SD 11.9) with a mean RHR_C_ of 51.3% (SD 10.2) as shown in Table 3. We found a mean ${\overset.{\mathrm V}\mathrm O}_{2,\mathrm{shift}}$ of 1267 ml/min (SD 161), which is on average 75.4% (SD 18.5) of the subjects VT1 or a RAS_shift_ of 49.4%${\overset.{\mathrm V}\mathrm O}_{2,\max}$ (SD 9.3). The mean ${\overset.{\mathrm V}\mathrm O}_{2,\mathrm C}$ with 1599 ml/min (SD 206) is 95.1% (SD 22.7) of the subjects VT1 or 62.8%${\overset.{\mathrm V}\mathrm O}_{2,\max}$ (SD 11). Further details are shown in Table 4. The calculated limits according to Wu & Wang (2002) for RHR_shift,rec_ range from 19.9% to 28.3% and for RHR_C,rec_ from 34.7% to 53.8%. RAS_shift,rec_ ranges from 30.9% to 36.5% and RAS_C,rec_ from 40.7% to 53.3% (Additional file 1: Recommended relative heart rate and aerobic strain) [17]. In general, heart rate and aerobic strain were higher in the field than these limits for both the whole shift (RHR_shift_, RAS_shift_) and the time expended collecting (RHR_C_, RAS_C_) as shown by plotting measured parameters against the calculated limit (Fig. 2). The mean differences were statistically significant (Table 5). For most of the participants, oxygen consumption in the field remained below the individual ventilatory threshold $({\overset.{\mathrm V}\mathrm O}_{2,\mathrm{VT}1})$ as shown by the plot in Fig. 2. The difference for the whole shift was statistically significant (${\overset.{\mathrm V}\mathrm O}_{2,\mathrm{shift}}-{\overset.{\mathrm V}\mathrm O}_{2,\mathrm{VT}1}=-491$ ml/min SD 382, *p* = 0.0015). Only the strain during the main task of manual collection of bulky waste $({\overset.{\mathrm V}\mathrm O}_{2,\mathrm C})$ did not show a significant difference to $({\overset.{\mathrm V}\mathrm O}_{2,\mathrm C}-{\overset.{\mathrm V}\mathrm O}_{2,\mathrm{VT}1}=-159\;\mathrm{ml}/\min\;\mathrm{SD}\;390,\;p\;=0.152)$.

**Table 3:** Results of time and of heart rate (HR) measurements in the field

***n* = 14**		**mean**	**SD**	**median**	**min**	**max**
**T**_**shift**_	[hours]	7.67	0.99	7.25	6.68	10.03
**T**_**C**_	[hours]	3.17	0.75	3.05	1.97	4.92
**T**_**driving**_	[hours]	3.95	0.75	3.77	3.12	5.45
**T**_**int,driving**_	[minutes]	14.8	4.1	9.1	1	81
**T**_**int,C**_	[minutes]	16.4	4.0	15.7	1	90
**T**_**breaks**_	[minutes]	33.5	13.5	31	7	50
**HR**_**shift**_	[bpm]	102	10.2	102	79.9	118
**HR**_**shift**_**%HR**_**VT1**_	[%]	85.7	10.6	85.3	62.9	102
**RHR**_**shift**_	[%]	36.9	8.0	37.0	23.6	51.9
**HR**_**C**_	[bpm]	116	11.9	118	92.6	134
**RHR**_**C**_	[%]	51.3	10.2	50.4	32.5	71.4

*T*_*shift*_ Individual shift in hours, *T*_*C*_ Individual total time of the main task of collecting manually bulky waste from the premises of the customer summed in hours, *T*_*driving*_ individual total time driving from customer to customer summed over T_shift_ in hours, *T*_*int,driving*_ Intervals of driving intervals between collection points, *T*_*int,C*_ Intervals of handling bulky waste, *T*_*breaks*_ Formal breaks from work, *HR*_*shift*_ Individual mean HR during the individual shift in hours (T_shift_), *RHR*_*shift*_ Individual mean relative heart rate (RHR) during T_shift_, *HR*_*C*_ Individual mean HR during the main task of collecting manually bulky waste from the premises of the customer summed in hours (T_C_), *RHR*_*C*_ Individual RHR during T_C_, *SD* Standard deviation

**Table 4:** Results of the calculated oxygen uptake in the field

***n* = 14**		**mean**	**SD**	**median**	**min**	**max**
${\overset{\boldsymbol.}{\mathbf V}\mathbf O}_{\mathbf2\boldsymbol,\mathbf s\mathbf h\mathbf i\mathbf f\mathbf t}$	[ml/min]	1267	161	1235	954	1539
${\overset{\boldsymbol.}{\mathbf V}\mathbf O}_{\mathbf2\boldsymbol,\mathbf s\mathbf h\mathbf i\mathbf f\mathbf t}\boldsymbol\%{\overset{\boldsymbol.}{\mathbf V}\mathbf O}_{\mathbf2\boldsymbol,\mathbf V\mathbf T\mathbf1}$	[%]	75.4	18.5	72	28.4	120
**RAS**_**shift**_	[%V̇O_2,max_]	49.4	9.3	48.5	36.6	65.0
${\overset{\boldsymbol.}{\mathbf V}\mathbf O}_{\mathbf2\boldsymbol,\mathbf C}$	[ml/min]	1599	206	1548	1348	2000
${\overset{\boldsymbol.}{\mathbf V}\mathbf O}_{\mathbf2\boldsymbol,\mathbf C}\boldsymbol\%{\overset{\boldsymbol.}{\mathbf V}\mathbf O}_{\mathbf2\boldsymbol,\mathbf V\mathbf T\mathbf1}$	[%]	95.1	22.7	91	62.8	146
**RAS**_**C**_	[%V̇O_2,max_]	62.8	11	65	43.1	82.2

${\overset.VO}_{2,shift}$ Individual mean $\mathrm{\overset{.}{V}O}_2$ during T_shift_, ${\overset.VO}_{2,VT1}$ Individual ventilatory threshold, *RAS*_*shift*_ Individual mean relative aerobic strain (RAS) during T_*shift*_, ${\dot{V}O}_{2,C}$ Individual mean $\mathrm{\overset{.}{V}O}_2$ during T_C_, *RAS*_*C*_ Individual RAS during T_C_, *SD* Standard deviation

**Table 5:** The differences between markers of physical strain and the recommended limits for prolonged occupational work [3, 17]

***n* = 14**		**mean**	**SD**	**median**	**min**	**max**	p^*^
**RHR**_**shift**_**-RHR**_**shift,rec**_	[%HRR]	11.2	8.7	10	-3.2	27.4	0.0014
**RHR**_**C**_**-RHR**_**C,rec**_	[%HRR]	6.9	9.4	5.2	-8.9	26.6	0.034
**RAS**_**shift**_**-RAS**_**shift,rec**_	[%V̇O_2,max_]	14.7	9.5	12.8	1.2	30.4	0.0004
**RAS**_**C**_**-RAS**_**C,rec**_	[%V̇O_2,max_]	15.7	10.2	17	0.6	30.9	0.0004
${\overset{\boldsymbol.}{\mathbf V}\mathbf O}_{\mathbf2\boldsymbol,\mathbf s\mathbf h\mathbf i\mathbf f\mathbf t}\boldsymbol-{\overset{\boldsymbol.}{\mathbf V}\mathbf O}_{\mathbf2\boldsymbol,\mathbf V\mathbf T\mathbf1}$	[ml/min]	-491	382	-482	-1299	196	0.0015
${\overset{\boldsymbol.}{\mathbf V}\mathbf O}_{\mathbf2\boldsymbol,\mathbf C}\boldsymbol-{\overset{\boldsymbol.}{\mathbf V}\mathbf O}_{\mathbf2\boldsymbol,\mathbf V\mathbf T\mathbf1}$	[ml/min]	-159	390	-140	-936	493	0.152

*RHR*_*shift*_ Individual mean relative heart rate (RHR) during T_shift_, *RHR*_*shift,rec*_ The calculated maximum recommended RHR for T_shift_ [17], *RHR*_*C*_ Individual RHR during T_C_, *RHR*_*C,rec*_ The calculated maximum recommended RHR for T_C_ [17], *RAS*_*shift*_ Individual mean relative aerobic strain (RAS) during T_shift_, *RAS*_*shift,rec*_ The calculated maximum recommended RAS for T_shift_ [17], *RAS*_*C*_ Individual RAS during T_C_, *RAS*_*C,rec*_ The calculated maximum recommended RAS for T_C_ [17], ${\overset.VO}_{2,shift}$ Individual mean oxygen consumption $(\mathrm{\overset{.}{V}O}_2)$ during the individual shift, ${\overset.VO}_{2,VT1}$ Individual oxygen consumption at ventilatory threshold (VT1), ${\dot{V}O}_{2,C}$ Individual mean $\mathrm{\overset{.}{V}O}_2$ during the individual total time collecting manually bulky waste from the premises of the customer summed over the duration of T_shift_ (T_C_), *SD* Standard deviation

^*^The shown *p*-values are adjusted for multiple comparisons with the Bonferroni-Holm method [37]

## Discussion

This field-study focuses on physical workload in relation to cardiopulmonary capacity, specifically among bulk waste collectors. Heart rate measurements and their correlation with individual oxygen consumption $(\mathrm{\overset{.}{V}O}_2)$ were used to assess the strain. The average oxygen uptake during the shift $({\overset.{\mathrm V}\mathrm O}_{2,\mathrm{shift}})$ was found to be 1267 ml/min, confirming the heavy physical workload of this occupation [16].

Our results show that most of the recommendations for RHR and RAS from the literature (Additional file 2: Acceptable limits of cardiovascular strain) for an acceptable workload were exceeded by the subjects. The limits based on $\mathrm{\overset{.}{V}O}_2$ at the ventilatory threshold 1 (VT1) of the individual subject were not exceeded (Tables 3, 4, 5, and Additional file 2: Acceptable limits of cardiovascular strain). This disparity could be explained by the athletic endurance capacity of the study participants with a high oxygen consumption at VT1 (${\overset.{\mathrm V}\mathrm O}_{2,\mathrm V\mathrm T1}\%{\overset.{\mathrm V}\mathrm O}_{2,\max,\mathrm{pred}}$ of 61.2% (SD 14.1)) (Table 2) [32]. These findings support VT1 being a better choice or the upper limit for heavy occupational work compared to relative heart rate (RHR) or relative aerobic strain (RAS), because ${\overset.{\mathrm V}\mathrm O}_{2,\mathrm{VT}1}$ takes the individual endurance capacity into account. RHR or RAS consider only the maximum work capacity (%HRR, $\%{\overset.{\mathrm V}\mathrm O}_{2,\max}$). For these parameters, it is assumed that the endurance capacity is 33% RHR [8], 40% RAS [10] or 40–45% RAS [38] as fixed percentages of the maximum work capacity, which should not be exceeded. The increased endurance capacity of subjects in hard-working occupations is not considered here. Therefore, the authors recommend using the HR or $\mathrm{\overset{.}{V}O}_2$ at the ventilatory threshold 1 (VT1) of the individual, which is the upper physiological limit to sustain prolonged physical work.

In the field measurements, the subjects were able to perform a total of 3.17 h of heavy occupational work, distributed over intervals (T_int,C_) of 16.4 min (SD 4) in the shift. During these intervals of collection, the oxygen consumption ${\overset.{\mathrm V}\mathrm O}_{2,\mathrm C}$ showed no differences from ${\overset.{\mathrm V}\mathrm O}_{2,\mathrm{VT}1}$ (*p* = 0.152) (Tables 4 and 5). This demonstrates, that a workload beyond VT1 for an extended duration is not feasible [24, 32].

Rapid slowing down of the HR was observed while driving (T_int,driving_) to the next customer, which took on average 14.8 min (SD 4.1) (Additional file 4: Heartrate measurements in the field). Therefore, these driving intervals can be counted as recovery phases. Resting for at least 5 min is described as sufficient recovery for the cardiopulmonary system [39–41]. These interruptions were sufficient to reduce the total strain during the shift below VT1. Ilmarinen's recommendation (1992) that intensive work over an 8-h workday is acceptable if breaks are available can be confirmed with these observations [14]. This is further confirmed by the findings of Moser et al. (2015), who indicated that there are no discernible differences in physiological reactions between continuous and high-intensity interval workloads [42].

Compared to previous studies conducted in waste management and occupations with high physical exertion, we found no significant differences in average heart rate during 8-h shifts (HR_shift_) in our collective (Additional file 3: Other studies vs. our results) [3–6, 13, 20]. We found lower cardiopulmonary strain compared to other studies in Brazil, the Netherlands, Iran, and Japan regarding HR, RHR, and RAS during the actual waste collection activity [4–6, 13]. The different work organisation, with smaller individual weights and other shift lengths, could be causal for these observed differences.

However, other studies measuring oxygen consumption $(\mathrm{\overset{.}{V}O}_2)$ showed significantly lower values during uninterrupted work compared to ${\overset.{\mathrm V}\mathrm O}_{2,\mathrm C}$ in our study, i.e., oxygen consumption only during the time with physical stress (T_C_) [3, 20]. These results suggest that bulk waste collection involves either higher peak workloads or higher total workloads compared to the aforementioned studies.

Comparing the oxygen consumption $(\mathrm{\overset{.}{V}O}_2)$ of garbage collectors [3] with our collective bulk waste collection shows a higher $\mathrm{\overset{.}{V}O}_2$ during the work intervals. This refers to the total $\mathrm{\overset{.}{V}O}_2$ compared to $\mathrm{\overset{.}{V}O}_2$ and also to $\mathrm{\overset{.}{V}O}_2$ relative to ${\overset.{\mathrm V}\mathrm O}_{2,\mathrm{VT}1}$ (*p* =  < 0.0001, *p* = 0.0001, resp.; Additional file 3: Other studies vs. our results). The work of bulk waste collectors seems to be more stressful, at least during the intervals of bulk waste collection.

Another subgroup of waste collectors included in an investigation of a variety of occupations [20] showed a similar result with lower values for total $\mathrm{\overset{.}{V}O}_2$ and lower $\mathrm{\overset{.}{V}O}_2$ in relation to VT1 during continuous work (-599 ml/min, -27%, resp.; *p* =  < 0.0001 and *p* < 0.001, resp.). The authors, however, performed this measurement of $\mathrm{\overset{.}{V}O}_2$ only over 20 min to obtain a comparative value. To assess the physical strain for the whole shift (8 h), they used the average HR relative to the HR at lactate turning point 1 (HR_8h_%HR_LTP1_). LTP1 is physiologically equivalent to VT1; consistently, the corresponding parameter to HR_8h_%HR_LTP1_ is HR_shift_ relative to HR at VT1 (HR_shift_%HR_VT1_). We found no significant difference between these parameters based on LTP1 and VT1 (-5.8%, *p* = 0.0828; Additional file 3: Other studies vs. our results). In summary, bulk waste collectors show higher peak loads but similar overall strain compared to other occupations with high physical workloads.

Despite being exposed to high cardiovascular strain on a daily basis, the subjects occasionally reached heart rates close to their maximum heart rate (HR_max_) during the field measurements and they showed average performance in terms of maximum oxygen consumption $({\overset.{\mathrm V}\mathrm O}_{2,\max})$ and maximum power output (P_max [W]_) during CPX (Table 2). These results confirm the previously mentioned findings that physically demanding work has no training effect on maximum aerobic capacity [19, 32, 43, 44]. However, our results indicate that it might have an effect on endurance performance (${\overset.{\mathrm V}\mathrm O}_{2,\mathrm{VT}1}\%{\overset.{\mathrm V}\mathrm O}_{2,\max,\mathrm{pred}}$ of 61.2% (SD 14.1)) [32].

The difference of 13.5% between ${\overset.{\mathrm V}\mathrm O}_{2,\max}\%{\overset.{\mathrm V}\mathrm O}_{2,\max,\mathrm{pred}}$ (90.5 SD 13) and P_max_%P_max,pred_ (104% SD 14.4) was expected because the predicted values for P_max_ are older and lower than the predicted values for ${\overset.{\mathrm V}\mathrm O}_{2,\max}$. Apart from this, the relationship between power output and $\mathrm{\overset{.}{V}O}_2$ is linear [32].

### Limitations and strengths

The cumulative volume of compacted bulky waste examined in this study was 3.9 metric tons per shift, resembling the company's historical data from 2005 to 2018, with an average of 3.7 tons. Thus, the selected tours can be considered representative.

Field-studies with individuals who move unpredictably (from a research perspective) and perform heavy physical labour are complex; they require a high level of commitment from the individuals involved, as measurement devices may interfere with their habitual movements and pace of work, which pose a safety risk. Therefore, direct $\mathrm{\overset{.}{V}O}_2$ measurement with the mobile CPX device was not possible. The assessment of $\mathrm{\overset{.}{V}O}_2$ via heart rate is a good alternative but may over- or underestimate the actual oxygen consumption during work because HR is influenced by several factors [45]. The most important factor possibly resulting in an elevated HR could be temperature, especially heat. The study was conducted with an average dry bulb temperature of 19.4°Celsius (SD 5.86; 12–30.9°Celsius) and a mean relative humidity of 47.5% (SD 16.6; 25–82. For heavy occupational work, the optimal temperature and relative humidity are considered 17 °C (15 °C-21°C) and 50% (30%-70%) [46]. Considering these recommendations, temperature and relative humidity were within the ranges of optimal climate conditions, where a low influence on HR can be expected. However, during peak temperatures, such as the highest temperature of 30.9°Celsius in our study, it was shown that $\mathrm{\overset{.}{V}O}_2$ may be overestimated up to 64% [40].

Medications that influence the HR were an exclusion criterion. No cardiovascular disease, metabolic disease, or psychiatric disease that could influence the HR were found in PME. Of the study population, 35.7% were overweight (BMI ≥ 25) and 42.9% were obese (BMI ≥ 30), which could result in a higher HR. In this study, five subjects smoked, which may have raised HR. Mental tension and noise due to the working conditions could not be ruled out and may have influenced HR. The influence of circadian rhythm is not relevant because we investigated all subjects at the same time of day. Even considering the possible overestimation of $\mathrm{\overset{.}{V}O}_2$, the feasibility of using HR measurement outweighs the health and safety risks of using devices for direct $\mathrm{\overset{.}{V}O}_2$ measurement.

The 14 subjects finally included in the analysis were on average younger than the baseline population (mean 42.6 years (25.6–57.1) (*n* = 14) vs. 47.3 (23–63) years (*n* = 105)) (Additional file 5: Characteristics of baseline population). This could lead to an overestimation of the mean ${\overset.{\mathrm V}\mathrm O}_{2,\max}$, as it tends to decrease with advancing age [38].

The study was conducted only once with each subject; therefore, intraindividual variations in performance may go unnoticed. Since the subjects were accompanied the whole time during the field measurements, it is possible that they may have changed their natural behaviour. Participating in a study and being under observation are possibly visible in the outliers of ${\overset.{\mathrm V}\mathrm O}_{2,\mathrm C}\%{\overset.{\mathrm V}\mathrm O}_{2,\mathrm{VT}1}$ with the lowest working at 62.8% and the highest at 146%.

## Conclusion

We found evidence that bulk waste collection is a heavy occupational activity comparable to other types of waste collection. Although the workload from the main task of lifting and carrying bulky waste is very high (at VT1 for more than 3 h), interruptions from other tasks or formal breaks distribute the load throughout the entire shift so that the total workload remains below the endurance limits (VT1). Most workers evidently choose a pace that matches their individual abilities. Again, using VT1 has been shown to be more appropriate as an individual limit for prolonged occupational exposure than commonly used blanket limits such as RAS and RHR.

### Supplementary Information

**Additional file 1.** Recommended relative heart rate and aerobic strain limits for Tshift and TC according to Wu & Wang [17].

**Additional file 2.** Acceptable limits of cardiovascular strain and corresponding parameters in our study.

**Additional file 3.** Mean value differences: Other studies vs. our results.

**Additional file 4.** Heartrate measurements in the field.

**Additional file 5.** Characteristics of baseline population compared to our study group.

## Data Availability

The dataset supporting the findings of this paper is available from the corresponding author on reasonable request.

## Glossary

BW: Body weight
CPX: Cardiopulmonary exercise testing
FEV1: Forced expiratory capacity in one second
FEV1_%pred_: % of the predicted FEV1
FVC: Forced vital capacity
FVC_%pred_: % of the predicted FVC
HR_8h_: Mean HR during an 8 h shift [20]
HR_8h_ %HR_LTP1_: HR_8h_ relative to the individual HR at the lactate turning point 1 (LTP1) [20]
HR_C_: Individual mean HR during T_C_
HR_driving_: Individual mean HR during T_driving_
HR_field_: HR average for each minute of the collection tour
HR_max_: Maximum heart rate
HR_max_ %HR_max,pred_: HRmax relative to predicted values of maximum heart rate (HR_max,pred_)
HR_SH_: Mean HR during the shift [3]
HR_shift_: Individual mean HR during T_shift_
HR_TGC_: Mean HR during TGC [4]
HR_PU bags_: Mean HR when working with polyurethane bags (PU bags) [13]
HR_trash cans_: Mean HR when working with metal trash cans [13]
HR_TWH_: Mean HR during TWH [4]
HR_VT1_: Heart rate at VT1
HR_VT1_%HR_max_: HR_VT1_ relative to HR_max_
HR_working_: Mean HR during a working day [5]
P: Power output during CPX
Parameter: A variable that describes a performance property or an aspect of that property
P_max_: Maximum power output during CPX
P_max_%P_max,pred_: P_max_ relative to predicted values of maximum power output (P_max,pred_)
P_max_/BW: P_max_ relative to the BW
P_VT1_: Power output at the ventilatory threshold 1 (VT1)
P_VT1_%P_max_: P_VT1_ relative to P_max_
RAS: Relative aerobic strain defined as the oxygen consumption (V̇O_2_) during work relative to the maximum possible V̇O_2_ in %V̇O_2,max_
RAS_1h_: Mean RAS during 1 h of work with direct V̇O_2_measurements [3]
RAS_20_: RAS during 20 minutes of work with direct V̇O_2_measurements [20]
RAS_8h, lifting_: Maximum acceptable RAS over an 8 h shift [ 15]
RAS_8h,shift,a_: Maximum acceptable RAS over an 8 h shift [13]
RAS_8h,shift,b_: Maximum acceptable RAS over an 8 h shift without sufficient break [14]
RAS_8h,shift,c_: Maximum acceptable RAS over an 8 h shift with sufficient breaks [14]
RAS_C_: Individual RAS during T_C_
RAS_C,rec_: The calculated maximum recommended RAS for T_C_ [17]
RAS_driving_: Individual mean RAS during T_driving_
RAS_field_: Relative aerobic strain calculated from _V̇O2,field_
RAS_shift_: Individual mean RAS during T_shift_
RAS_shift,rec_: The calculated maximum recommended RAS for T_shift_ [17]
RAS_PU bags_: Mean relative aerobic strain (RAS) when working with poly urethane (PU) bags [13]
RAS_trash cans_: Mean relative aerobic strain (RAS) when working with metal trash cans [13]
RER_max_: Maximum respiratory exchange rate
RHR: Relative heart rate
RHR_8h,shift_: Maximum acceptable RHR over an 8 h shift [8]
RHR_C_: Individual RHR during T_C_
RHR_C,rec_: The calculated maximum recommended RHR for T_C_ [17]
RHR_driving_: Individual mean RHR during T_driving_
RHR_field_: HR_field_ relative to the individual heart-rate-reserve in percent
RHR_SH_: Mean RHR during the shift [3]
RHR_shift_: Individual mean RHR during T_shift_
RHR_shift,rec_: The calculated maximum recommended RHR for T_shift_ [17]
RHR_TGC_: Mean relative HR (RHR) during TGC [4]
RHR_TWH_: Mean relative HR (RHR) during TWH [4]
RHR_working_: Mean relative HR during a working day [5]
SD: Standard deviation
sR_tot_: Total specific airway Resistance
T_C_: Individual total time of the main task of collecting manually bulky waste from the premises of the customer summed in hours
T_driving_: Individual total time driving from customer to customer summed over T_shift_ in hours
Tiffeneau-Index: (FEV1/FVC)x100%
T_int,C_: Duration of intervals of handling bulky waste at the customers
T_int,driving_: Duration of driving intervals between collection points
TLC: Total lung capacity
TLC_%pred_: % of the predicted TLC
T_shift_: Individual shift in hours
T_test phase_: Duration of pedaling with increasing load during CPX
TGC: Time of effective garbage collection [4],
TWH: Total working hours [4],
VT1: Ventilatory threshold 1 [23]
${\overset.{\mathrm V}\mathrm O}_{2,1\mathrm h}$: Mean oxygen consumption ($\mathrm{\overset{.}{V}O}_2$) during 1 h of work with direct $\mathrm{\overset{.}{V}O}_2$measurements [3]
${\overset.{\mathrm V}\mathrm O}_{2,1\mathrm h}\%{\overset.{\mathrm V}\mathrm O}_{2,\mathrm{VT}1}$: ${\overset.{\mathrm V}\mathrm O}_{2,1\mathrm h}$ relative to the individual $\mathrm{\overset{.}{V}O}_2$ at the ventilatory threshold 1 (VT1) [3]
${\overset.{\mathrm V}\mathrm O}_{2\;20}$: Mean $\mathrm{\overset{.}{V}O}_2$ during 20 minutes of work with direct $\mathrm{\overset{.}{V}O}_2$ measurements [20]
${\overset.{\mathrm V}\mathrm O}_{2\;20}\%{\overset.{\mathrm V}\mathrm O}_{2,\mathrm{LTP}1}$: ${\overset.{\mathrm V}\mathrm O}_{2\;20}$ relative to the individual $\mathrm{\overset{.}{V}O}_2$ at LTP1 [20]
${\overset.{\mathrm V}\mathrm O}_{2,8\mathrm h,\mathrm{shift}}$: Maximum acceptable oxygen consumption per minute over an 8 h workday [3, 18]
${\overset.{\mathrm V}\mathrm O}_{2,\mathrm C}$: Individual mean $\mathrm{\overset{.}{V}O}_2$ during T_C_
${\overset.{\mathrm V}\mathrm O}_{2,\mathrm{driving}}$: Individual mean $\mathrm{\overset{.}{V}O}_2$ during T_driving_
${\overset.{\mathrm V}\mathrm O}_{2,\mathrm{field}}$: Calculated oxygen consumption from HR_field_ according to [3, 17, 32, 33]
${\overset.{\mathrm V}\mathrm O}_{2,\max}$: Maximum oxygen consumption
${\overset.{\mathrm V}\mathrm O}_{2,\text{max}}\%\mathrm{BW}$: ${\overset.{\mathrm V}\mathrm O}_{2,\max}$ relative to the BW
${\overset.{\mathrm V}\mathrm O}_{2,\max}\%{\overset.{\mathrm V}\mathrm O}_{2,\max,\mathrm{pred}}$: ${\overset.{\mathrm V}\mathrm O}_{2,\max}$relative to predicted values of maximum oxygen consumption (${\overset.{\mathrm V}\mathrm O}_{2,\max,\mathrm{pred}}$)
${\overset.{\mathrm V}\mathrm O}_{2,\mathrm{shift}}$: Individual mean ${\dot{\text{V}}\text{O}}_{2}$ during T _shift_
${\overset.{\mathrm V}\mathrm O}_{2,\mathrm{VT}1}$: Oxygen consumption at the VT1
${\overset.{\mathrm V}\mathrm O}_{2,\mathrm{VT}1}\%{\overset.{\mathrm V}\mathrm O}_{2,\max,\mathrm{pred}}$: ${\overset.{\mathrm V}\mathrm O}_{2,\mathrm{VT}1}$ relative to ${\overset.{\mathrm V}\mathrm O}_{2,\max,\mathrm{pred}}$ as a marker for endurance capacity according to [32]
