# Supplementary figures and images for: Physical workload and cardiopulmonary parameters in relation to individual capacity of bulk waste workers – a cross-sectional field-study
Source: J Occup Med Toxicol. 2023 Dec 15;18:29. doi: 10.1186/s12995-023-00389-z (PMC10724959; doi:10.1186/s12995-023-00389-z)

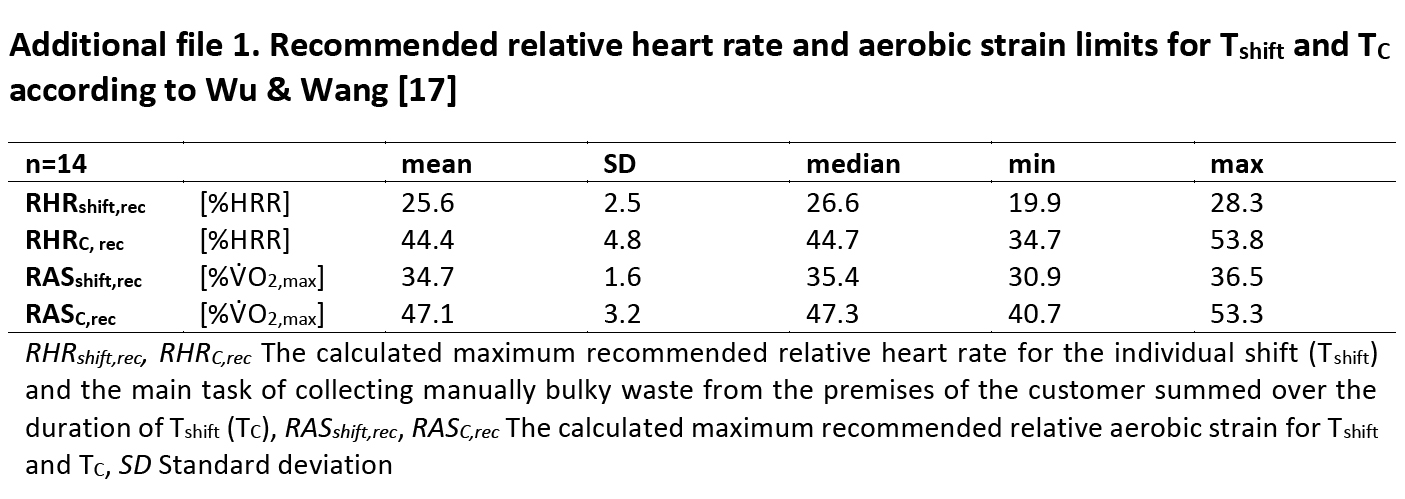

Supplement: Supplementary file 1 — Additional file 1. Recommended relative heart rate and aerobic strain limits for Tshift and TC according to Wu & Wang [17]. [file 12995_2023_389_MOESM1_ESM.jpg]

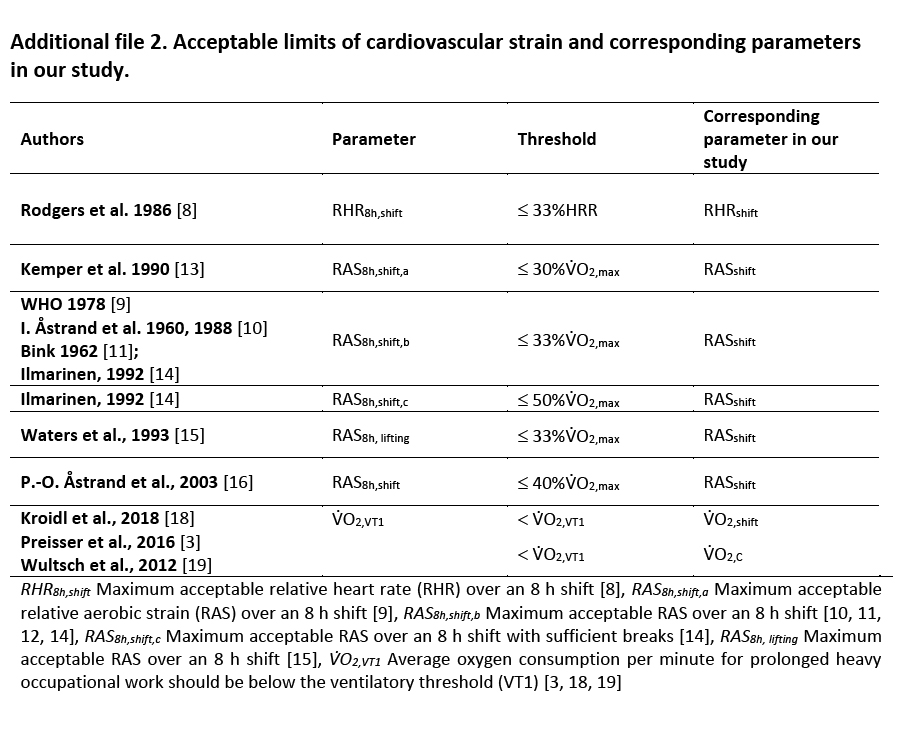

Supplement: Supplementary file 2 — Additional file 2. Acceptable limits of cardiovascular strain and corresponding parameters in our study. [file 12995_2023_389_MOESM2_ESM.jpg]

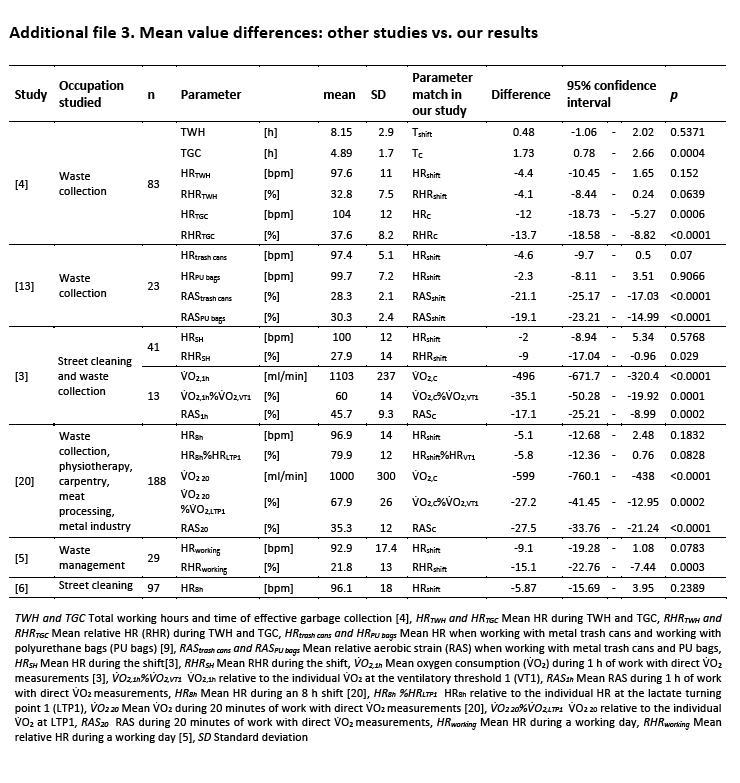

Supplement: Supplementary file 3 — Additional file 3. Mean value differences: Other studies vs. our results. [file 12995_2023_389_MOESM3_ESM.jpg]

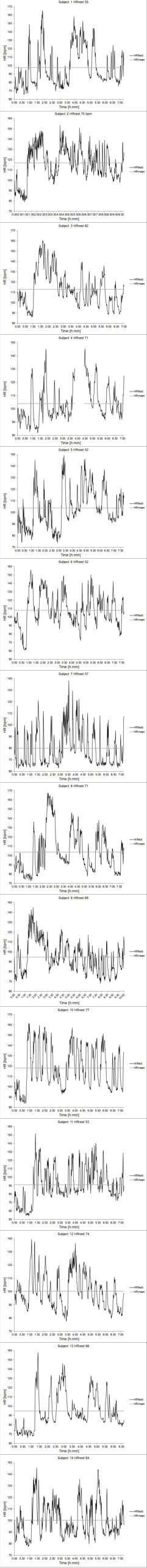

Supplement: Supplementary file 4 — Additional file 4. Heartrate measurements in the field. [file 12995_2023_389_MOESM4_ESM.jpg]

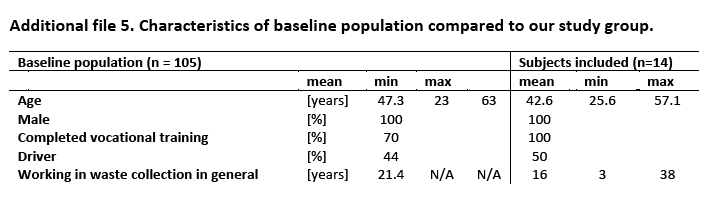

Supplement: Supplementary file 5 — Additional file 5. Characteristics of baseline population compared to our study group. [file 12995_2023_389_MOESM5_ESM.jpg]
